# 

**Published:** 1867-12

**Authors:** 


Vol. V. *November & December*, 1867. No. 1.

THE

### IOWA MEDICAL JOURNAL,

EDITED BY

J. C. HUGHES, M. D.,

*Professor of the Institutes, and Practice of Surgery and Surgical Clinics in the Medical Department Iowa State University; Surgeon-in-Charge of the College Infirmary and Eye and Ear Institute; Member of American Medical Association and of the British Association for the Promotion of Science, etc., etc.*

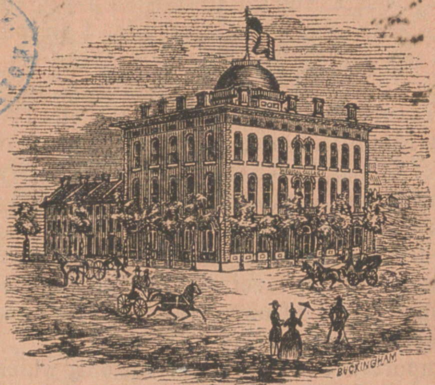

KEOKUK, IOWA.

Published every alternate Month.

Price, Two Dollars a Year, in advance.

Editors and Publishers Please Exchange.

Surgeon Gen'l's Office LIBRARY. 14728 Washington, D.C.

14798

Address all Communications, Periodicals or Books to the Editor.

*KEOKUK, IOWA:*
PRINTED AT THE GATE CITY BOOK AND JOB ROOMS.
1867.

Letters requiring an answer, must contain a Stamp to insure attention.

